# Collecting routine and timely cancer stage at diagnosis by implementing a cancer staging tiered framework: the Western Australian Cancer Registry experience

**DOI:** 10.1186/s12913-024-11224-4

**Published:** 2024-06-28

**Authors:** Shantelle J. Smith, Rachael Moorin, Karen Taylor, Jade Newton, Stephanie Smith

**Affiliations:** 1 School of Population Health, Curtin University, Perth, WA Australia; 2 Faculty of Health Sciences, Curtin Health Innovation Research Institute, Curtin University, Bentley, WA Australia; 3 School of Population and Global Health, The University of Western Australia, Crawley, WA Australia; 4 Cancer Network WA, North Metropolitan Health Service, Perth, WA Australia; 5 Curtin Medical School, Curtin University, Perth, WA Australia

**Keywords:** Cancer staging tiered framework, Population-based cancer staging, Cancer registry, Cancer staging at diagnosis, Cancer staging methods, Population-based cancer registries

## Abstract

**Background:**

Current processes collecting cancer stage data in population-based cancer registries (PBCRs) lack standardisation, resulting in difficulty utilising diverse data sources and incomplete, low-quality data. Implementing a cancer staging tiered framework aims to improve stage collection and facilitate inter-PBCR benchmarking.

**Objective:**

Demonstrate the application of a cancer staging tiered framework in the Western Australian Cancer Staging Project to establish a standardised method for collecting cancer stage at diagnosis data in PBCRs.

**Methods:**

The tiered framework, developed in collaboration with a Project Advisory Group and applied to breast, colorectal, and melanoma cancers, provides business rules – procedures for stage collection. Tier 1 represents the highest staging level, involving complete American Joint Committee on Cancer (AJCC) tumour–node–metastasis (TNM) data collection and other critical staging information. Tier 2 (registry-derived stage) relies on supplementary data, including hospital admission data, to make assumptions based on data availability. Tier 3 (pathology stage) solely uses pathology reports.

**Findings:**

The tiered framework promotes flexible utilisation of staging data, recognising various levels of data completeness. Tier 1 is suitable for all purposes, including clinical and epidemiological applications. Tiers 2 and 3 are recommended for epidemiological analysis alone. Lower tiers provide valuable insights into disease patterns, risk factors, and overall disease burden for public health planning and policy decisions. Capture of staging at each tier depends on data availability, with potential shifts to higher tiers as new data sources are acquired.

**Conclusions:**

The tiered framework offers a dynamic approach for PBCRs to record stage at diagnosis, promoting consistency in population-level staging data and enabling practical use for benchmarking across jurisdictions, public health planning, policy development, epidemiological analyses, and assessing cancer outcomes. Evolution with staging classifications and data variable changes will futureproof the tiered framework. Its adaptability fosters continuous refinement of data collection processes and encourages improvements in data quality.

**Supplementary Information:**

The online version contains supplementary material available at 10.1186/s12913-024-11224-4.

## Introduction

Cancer stage at diagnosis informs the healthcare team of the patient’s prognosis and aids in determining the most effective treatment approach [1]. It describes the extent or spread of cancer at the initial diagnosis and after staging investigations for distant disease before any treatment has been delivered. Population-level collection of staging data can guide health service planning and evaluate cancer control and early detection initiatives [2]. When linked with other national and international data sources, it can explore stage-specific cancer outcomes, geographic and socioeconomic variation, and survival [3].

Our recent scoping review determining cancer stage in population-based cancer registries (PBCRs) identified three categories of staging classification systems for assigning stage: 1) tumour–node–metastasis (TNM)-based, 2) categorisation by local, regional and distant spread, and 3) miscellaneous systems [4]. In Australian clinical practice, cancer stage is classified primarily using the most widely used American Joint Committee on Cancer (AJCC) 8th edition TNM staging classification system for solid tumours [4, 5]. The TNM classification describes the extent of the primary tumour (T category), the involvement of nearby lymph nodes (N category), and the presence or absence of distant metastasis (M category). Based on the findings of TNM and occasionally non-anatomic values (e.g., Gleason score and prostate-specific antigen level for prostate cancer), an overall stage group can be assigned, ranging from Stage I-IV [5]. To apply TNM categories accurately, certain staging rules and classifications are necessary, which involve considering the diagnosis date, the timeframe for staging, and utilising prefix stage classifications. The AJCC TNM staging system utilises prefixes including “p” for the pathological stage (pTNM), “c” for the clinical stage (cTNM), and “y” for the post-therapy stage (yTNM), which assists the PBCR in determining the stage at diagnosis [5].

Cancer staging information is typically documented in unstructured free-text format, dispersed across various sources, such as multidisciplinary team meeting notes, medical correspondence, hospital-based cancer databases, and pathology and radiology reports, instead of being stored in structured data fields [6]. This unstructured approach makes it challenging to systematically capture assessments of cancer stage in clearly defined data fields suitable for population-level analysis. Moreover, achieving a comprehensive assessment of the stage at diagnosis involves correlating data from multiple diagnostic tests and physician reviews, all of which must align with the staging classification system [5]. These individual pieces of staging information may be distributed across different medical records or locations, often spanning several weeks of clinical investigative processes.

Current approaches to the routine collection of cancer stage at diagnosis in PBCRs are constrained by the absence of standardised methodologies for collecting staging data, resulting in poor quality or incomplete data, and difficulties in accessing relevant data sources [4, 7, 8]. The collection of cancer stage at diagnosis in PBCRs has been found to encompass a variety of methods, relying on a wide range of data sources connected to routine data pipelines and collection processes – highlighting the diversity and complexity of how cancer stage information is gathered in PBCRs [4]. A recent study on staging data completeness for endometrial cancers in PBCRs in Australia in 2018 and 2019 highlights the significant variability and inconsistency across jurisdictions [9]. Only four of eight jurisdictions were capable of deriving AJCC TNM stage: using pathology reports alone, stage could be calculated in 6% of cases in Western Australia and 25% in the Northern Territory [9]. In Victoria, where both pathology reports and hospital admitted data were available, 88% of cases could be staged. Queensland was the only state with a variety of routinely available datasets – including pathology reports, hospital admitted data, multidisciplinary meeting (MDM) data, oncology information systems, and public radiology data – enabling 90% of cases to be staged [9]. South Australia had no stage data for those years, and the remaining three registries collected Degree of Spread stage classification [9].

Outside of Australia, efforts to benchmark cancer outcomes across countries revealed significant variability in staging data collected by different cancer registries, including differences in staging variables and sources of information [7]. In Northern Africa and the Middle East region, 23 PBCRs reported on their staging practices, with 21 collecting staging data using either a single classification system (14 registries using either TNM or Surveillance, Epidemiology and End Results (SEER) summary stage) or both (7 registries), but without specifying the sources of their staging data [10].

The utilisation of different staging classification systems creates challenges in achieving harmonisation and cross-jurisdictional comparisons, with staging conversion systems raising the risk of misclassification [4]. Consequently, reliance on multiple staging classifications or inconsistent staging data results in the use of incomparable cancer stage data, impacting research, clinical decision-making, and policy development [4]. The disparity in data quality blocks collaborative efforts due to the absence of standardised data for information amalgamation or comparative analyses [7]. In clinical decision-making based on PBCR data, challenges arise in providing data linkages to evaluate treatment efficacy or compliance with clinical guidelines due to inadequate staging data [11]. Additionally, it hinders policy development for benchmarking progress or assessing cancer control initiatives, making it difficult to assess the effectiveness of interventions and impeding efforts for continuous improvement [8, 11, 12]. As a result, policymakers face difficulties in allocating resources judiciously, potentially leading to misguided decisions and resource imbalances in specific areas [13]. This highlights the urgent need for standardising data collection processes.

The collection of cancer stage in PBCRs has not always been justified due to the substantial effort and time required for manual review and input, which has generally been the primary method for staging [1, 2]. This is challenging for PBCRs who often have limited financial and physical resources [3], such as digital health infrastructure and workforce, in addition to the added complication of evolving rules and guidelines in staging systems [14].

In Australia, many PBCRs typically do not collect or report cancer stage information, prioritising the collection of data needed to report incidence and mortality rates only (Australasian Association of Cancer Registries: Australian PBCR Staging Assessment, unpublished). The inability to meet the demand for cancer stage information to assess outcomes and evaluate healthcare at the population level for cancer control has been a long-standing concern [15, 16]. To investigate how to progress this unmet need, each Australian state and territory cancer registry is collaborating with Cancer Australia (a government agency established in 2006 to benefit all Australians affected by cancer) to scope out current collection methods and explore sustainable solutions for routine capture. The Staging, Treatment and Recurrence (STaR) project in 2015 was an early initiative aimed at collecting 2011 cancer stage data in Australian PBCRs [17].

### 2011 STaR project and current cancer staging approaches in Australia

The 2011 STaR project, piloted by Cancer Australia in collaboration with the Australian Institute of Health and Welfare (AIHW) and state and territory cancer registries, is the sole national-level initiative for gathering staging data [17]. The staging data only captured those diagnosed in 2011, and the data from this pilot remains the most recent available [8]. It aimed to improve cancer outcomes by providing consistent and accurate staging information to healthcare professionals, researchers, and patients [18]. However, achieving improved cancer outcomes with only one year of national staging data is not feasible, especially considering the closure of the project and the data being collected according to the AJCC TNM 7th edition (Australasian Association of Cancer Registries: Australian PBCR Staging Assessment, unpublished). This underscores the necessity for further staging efforts to effectively monitor cancer outcomes.

The STaR project required PBCRs to provide a registry-derived stage (RD-stage) for the top five highest-incidence cancers diagnosed in 2011 (prostate, breast, lung, colorectal and melanoma) [2, 17]. RD-stage was defined as the stage category at diagnosis obtained from notification sources routinely available to PBCRs and derived using simplified AJCC TNM business rules and algorithms developed by the Victorian Cancer Registry (VCR) [2]. Business rules were developed to articulate the decision-making process used to define each stage category and to align the data with the AJCC TNM standard, especially when assumptions are required due to lack of data [19]. Although the 2011 STaR project yielded nearly comprehensive national cancer staging information, with the exception of lung cancer – where almost one-third of staging data remained unknown – it required significant manual effort and training for registry coders to extract TNM data from the mandatory notification sources, as well as adequate resources for applying business rules [19]. Additionally, the time spent on deriving RD-stage impacted routine coding processes [2]; for example, in Western Australia (WA), participation was entirely dependent on short-term additional project funding which was not sustained.

Following the conclusion of the 2011 STaR project due to feasibility issues, only a few state and territory cancer registries have persevered in collecting staging information within the constraints of their data pipelines and available resources – Victoria was the only PBCR that continued with RD-stage business processes, continuing only for breast, colorectal and melanoma cancers (Australasian Association of Cancer Registries: Australian PBCR Staging Assessment, unpublished). This did not extend to lung or prostate cancer due to poor data completeness at a population level (~57%) for lung cancer and updates in the 8th edition of the AJCC staging manual for prostate cancer. Other PBCR approaches range from foundational efforts like manually collecting explicit pathological stage data (pTNM) from pathology reports, to developing data science techniques such as natural language processing (NLP) and machine learning (ML) to automate and facilitate extracting information from relevant data sources, reducing or eliminating manual intervention (Australasian Association of Cancer Registries: Australian PBCR Staging Assessment, unpublished). Advancements in text-mining methods, specifically NLP and ML techniques, have demonstrated their effectiveness in extracting unstructured, free-text clinical data (e.g., clinical notes, radiology reports, pathology reports) across numerous healthcare and medical domains. These applications include processing clinical notes for symptom information, developing case-detection algorithms for clinical conditions, and transforming clinical text for chronic diseases into structured data [20–23]. Notably, the extraction of cancer information from electronic health records (EHRs), including the classification of cancer staging from pathology reports, has also gained prominence, highlighting the utility and relevance of these techniques for cancer stage data collection [21, 24–29].

**Fig. 1:**
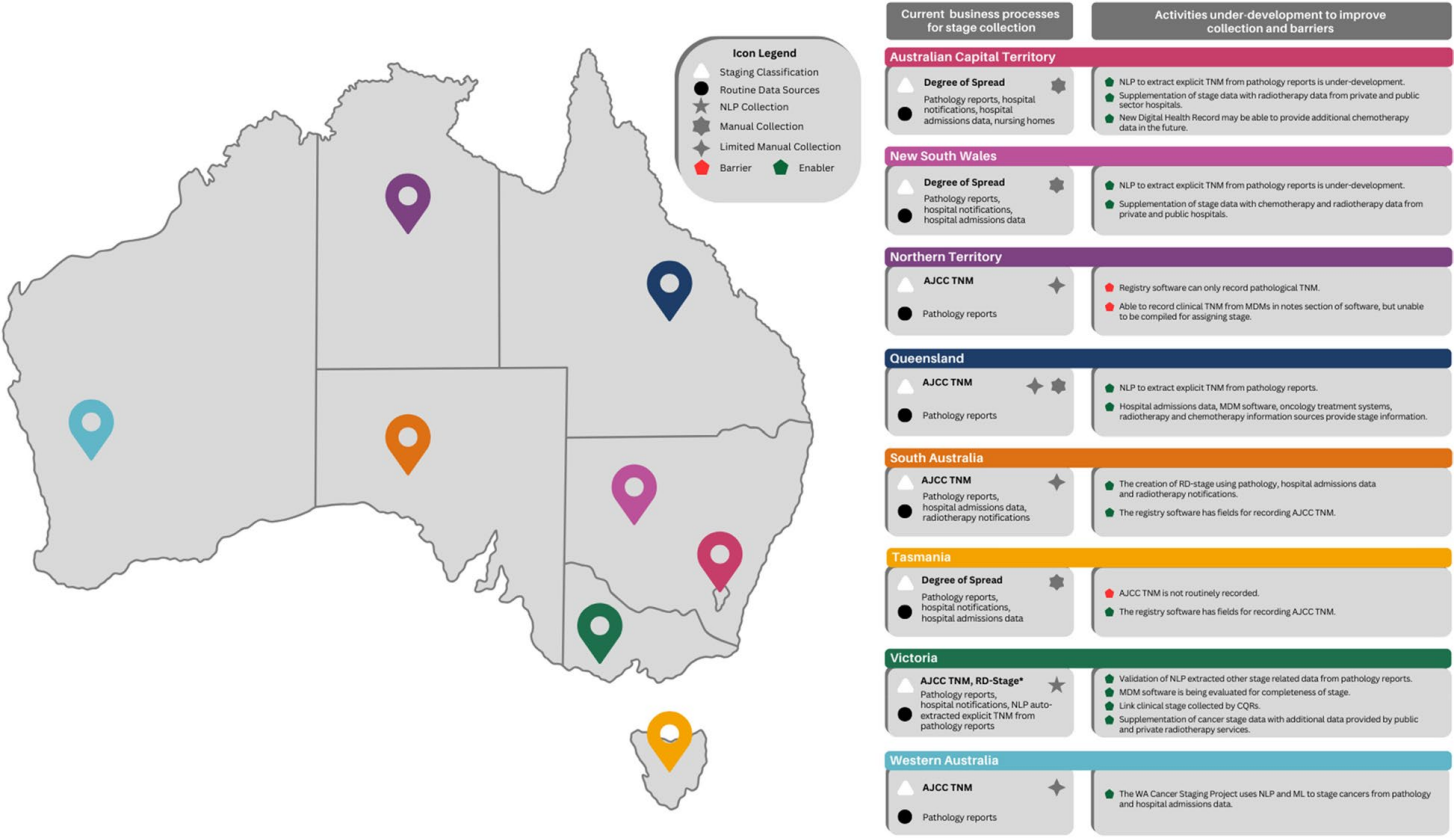
Collection of cancer stage in Australian Population-Based Cancer Registries (PBCRs). * Registry-Derived Stage is only collected for prostate, breast, colorectal and endometrial cancers, and melanoma. Notes: TNM – Tumour-Nodes-Metastasis; AJCC – American Joint Committee on Cancer Staging System; MDM – Multidisciplinary Team Meeting; NLP – Natural Language Processing; ML – Machine Learning; CQRs – Clinical Quality Registries

Figure 1 summarises the current operational business processes of each jurisdictional PBCR for routinely recording cancer stage, the activities that are currently under development by PBCRs, including NLP and ML extraction, as well as efforts and barriers to enhancing data availability (Australasian Association of Cancer Registries: Australian PBCR Staging Assessment, unpublished). The data depicted in this figure is sourced from the Australian PBCR Staging Assessment, conducted by the Australasian Association of Cancer Registries (AACR) and commissioned by Cancer Australia in 2023. This initiative aimed to investigate the stage collections of PBCRs in each state and territory, culminating in a report that provides recommendations for stakeholders to facilitate consistent and high-quality collection of stage at diagnosis (Australasian Association of Cancer Registries: Australian PBCR Staging Assessment, unpublished). The report noted that following the staging efforts of the STaR project and subsequent stage collection within the constraints of the PBCR, many states are currently in the process of developing NLP approaches to extract explicit TNM categories from pathology reports for automated stage collection (Australasian Association of Cancer Registries: Australian PBCR Staging Assessment, unpublished). However, this requires manual validation and funding, which may not be readily available to all PBCRs. The WA Cancer Registry (WACR) is developing NLP and ML techniques through the WA Cancer Staging Project, funded specifically for staging initiatives. To recognise the implications of utilising diverse data sources for staging, it has proposed a tiered framework for the ongoing collection of cancer stage at diagnosis.

### The need for a cancer staging tiered framework

Our recent research recommends the use of a tiered framework to standardise cancer stage collection, addressing variable data maturity levels among PBCRs throughout Australia [4, 8]. The tiered approach not only promotes data standardisation and comparability in registries using the AJCC TNM staging classification but also serves as an implementation strategy for capturing stage at diagnosis using existing data, allowing adjustments as data quality and completeness improve. The tiered framework proposes a systematic approach to collecting cancer stage data in registries based on data availability and should not be confused with the classification of stage. By enabling PBCRs to assess their data systematically, the framework prevents the comparison of incomparable data and recognises the variability in staging information.

## Aim

The aim of this paper is to demonstrate the application of a cancer staging tiered framework by the WA Cancer Staging Project in the WACR to establish a standardised method for collecting cancer stage information in PBCRs.

This paper does not adhere to a standard research format, and therefore its remaining structure is organised as follows: 1) Overview of the current approach to collecting cancer stage in the WACR, 2) Development of the cancer staging tiered framework, encompassing the business rules, 3) Application of the cancer staging tiered framework in the WACR to breast, colorectal and melanoma staging data. This is followed by discussions on the quality implications and appropriate use of staging data, the transition between tiers, and considerations for futureproofing the framework.

## Collecting cancer stage in the WACR

### WACR background

Since 1982, the WACR has provided data on cancer incidence, survival, and mortality for use in health service planning and cancer control evaluation, and to support cancer-related research [30]. The main sources of information to the WACR are reports from pathologists, haematologists, and radiation oncologists, supplemented by death registrations, hospital statistical discharge records, as well as information from hospital files and clinical information systems. The WACR collects detailed information on patient demographics, tumour-specific details, and diagnosis information. Each year, the WACR reports incidence and mortality data to the Australian Institute of Health and Welfare, where it undergoes further data cleaning and standardisation to produce the Australian Cancer Database, which includes data from all state and territory PBCRs [31].

### WA cancer staging project

In collaboration with the WACR, the Cancer Network WA has provided funding to Curtin University since June 2021 to support the WA Cancer Staging Project, which aims to develop and deliver statewide population-based staging in the registry. The project is establishing sustainable data collection methods, including NLP and ML algorithms, to decrease reliance on manual extraction. A Project Advisory Group (PAG) offers strategic advice and guidance to the project and oversees the expert tumour-specific clinical working groups that provide clinical expertise and assist with the development of business rules. Further information on the WA Cancer Staging Project has been published in our recent process evaluation, exploring key stakeholders’ perceptions of implementing cancer staging into the WACR [8]. The findings from our process evaluation highlighted major barriers to collecting cancer staging data, primarily stemming from a lack of standardisation and resulting in limited opportunities for benchmarking and fostering collaboration in cancer research and care.

### Collecting cancer stage

The WACR relies primarily on pathology data as the source of cancer incidence. Extent of disease (regional and distant involvement) information, often captured in radiology reports necessary for cancer stage, is not routinely notified to the WACR [30]. According to the legislation, radiology providers are not mandated to notify WACR of malignant radiology reports. In some instances, the WACR coding staff may import radiology reports sourced from clinical systems, where available, to ascertain diagnostic confirmation needed for incidence collection [30]. Starting in 2018, the WACR has opportunistically collected cancer staging data by manually extracting TNM information from pathology reports during routine coding. This data has been collected based on explicit reporting of TNM values within the pathology report and has not undergone validation, remaining incomplete in its capture. For example, only patients who undergo resection of their primary tumour will have pathological stage (pTNM) documented in their pathology report for WACR coding staff to collect. Consequently, there is a possibility of under-staging patients without additional clinical correlation to determine the extent of the disease. This approach results in the exclusion of patients who are not suitable for resection of the primary tumour, especially those with advanced disease.

To facilitate the routine and comprehensive collection of cancer stage in both WACR and other Australian PBCRs, steps must be taken including integration of additional data sources, implementation of staging procedures (business rules), and infrastructure reform. The capacity of the WACR to collect cancer staging within the routine coding process has been limited by the manual effort required, the need for trained personnel, the restricted data entry fields in the bespoke WACR database, and the incompleteness of cancer staging information due to the lack of access to radiology reports and other data sources (such as multidisciplinary team (MDT) meeting notes), as highlighted in our process evaluation [8].

To address these challenges in the WACR, the database and data collection tool will need to be enhanced to incorporate additional data fields capturing staging information and other important data elements from multiple sources, including coded hospital admitted patient data (known as the hospital morbidity data collection (HMDC) in WA), containing International Statistical Classification of Diseases and Related Health Problems, Tenth Revision, Australian Modification (ICD-10-AM) coding. During our process evaluation, a significant concern arose regarding the outdated WACR database’s ability to accommodate staging information [8]. Since updating the existing fields in the registry’s database is not currently possible, the WA Cancer Staging Project has created Research Electronic Data Capture (REDCap) platforms to store and manage all cancer staging information that is currently being collected [32]. In the absence of primary sources such as radiology reports (e.g. computed tomography (CT) scans, positron emission tomography (PET) scans, and magnetic resonance imaging (MRI)), the WACR must rely on secondary data, specifically the HMDC, to collect information on disease spread for staging purposes. All HMDC data elements are collected as individual variables in REDCap separate from TNM information obtained from primary sources (e.g., pathology reports). The HMDC data can complement primary sources and storing them individually enables assessment of dependence on secondary sources and how this reliance might evolve over time. The inclusion of secondary data in the routine collection process requires systematic review of all HMDC records that occur within a certain pre-specified time frame of the initial diagnosis date. The timing rule for HMDC collection inclusion was taken from the AJCC and 2011 STaR definitions for determining stage at diagnosis, which states 4 months (120 days) from the date of diagnosis as the window for staging data collection (Victorian Cancer Registry and Cancer Council Victoria: Definition of Registry Derived Stage and general TNM staging rules, unpublished) [5]. The time frame restriction is critical for accurately determining the extent of the disease prior to initiating first treatment and ensuring the most accurate estimation of TNM staging at diagnosis [5]. The WA Cancer Staging Project worked closely with the clinical working groups (and overseen by the PAG) to define business rules for utilising all data sources in cancer stage assignment. These rules cover various aspects, including: defining inclusion dates for primary and secondary data (e.g., 120 days from the date of diagnosis); determining priority through decision-tree logic (for instance, favouring more advanced TNM values in case of conflicting clinical reports); and allocating stages within the cancer staging tiered framework, as examples. These business rules were also heavily informed by those used in the 2011 STaR project.

## Developing the cancer staging tiered framework

**Fig. 2:**
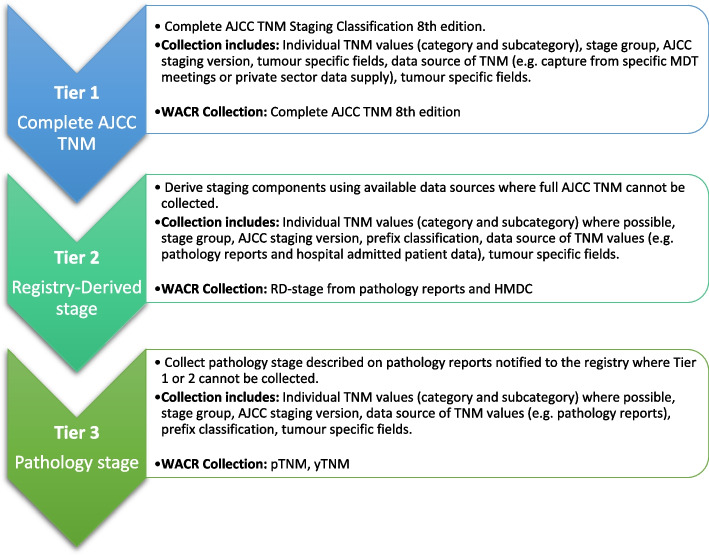
Cancer Staging Tiered Framework. Notes: TNM – Tumour, Nodes, Metastases; AJCC – American Joint Committee on Cancer Staging System; MDT – Multidisciplinary Team; WACR – Western Australian Cancer Registry; RD-stage – Registry-Derived stage; HMDC – Hospital Morbidity Data Collection; pTNM – Pathological stage; yTNM – Post-therapy stage

At the outset of the WA Cancer Staging Project, the tiered framework was developed to provide guidance and flexibility for the collection of cancer stage data. It acknowledged the diversity of stage data collection in Australia, emphasising that it is not a one-size-fits-all approach, and recognising that data restrictions are often encountered [4]. The tiered framework is a set of rules for collecting staging that incorporates different available data sources and presents an explicit hierarchy of completeness (Fig. 2).

The formulation of the tiered framework occurred during the conceptualisation and implementation phases of NLP and ML models for the breast and colorectal cancer streams within the WA Cancer Staging Project. Its design aimed to strategically align with efforts in data standardisation. Subsequently, the framework was later applied to the melanoma stream, broadening its applicability and impact for future cancer streams and other PBCRs.

Tier 1 (the gold standard) facilitates the complete AJCC TNM Staging Classification and provides staging information suitable for both epidemiological and clinical use. The lowest level (Tier 3) describes pathology derived stage using basic information available to all registries. While this tier is the least complex, and therefore most achievable, there is a significant risk of under-staging. Tier 2 provides a middle ground by incorporating available secondary data sources to partially fill the gap between complete AJCC TNM and pathology derived stage. The tiered framework was aimed at ensuring long-term data integrity, facilitating interoperability (i.e., explicit understanding of the level of staging) for sharing and collaborating using staged data, and, lastly, standardisation for stage categorisation and reporting across time and/or jurisdictions.

### Expert involvement

The cancer staging tiered framework was collaboratively developed with the WA Cancer Staging Project’s PAG, which included a range of expertise, including healthcare professionals and specialists, the Department of Health WA registry and coding staff, consumer representatives (patients with lived cancer experience), health researchers, and cancer organisations [8]. The development process involved iterative steps, including literature review, presentation of evidence at consultative meetings with the PAG, and incorporation of findings from our scoping review conducted early in the project [4]. The scoping review identified methods used for population-based stage collection and stage classification in PBCRs, including considering the strengths and disadvantages [4].

**Fig. 3:**
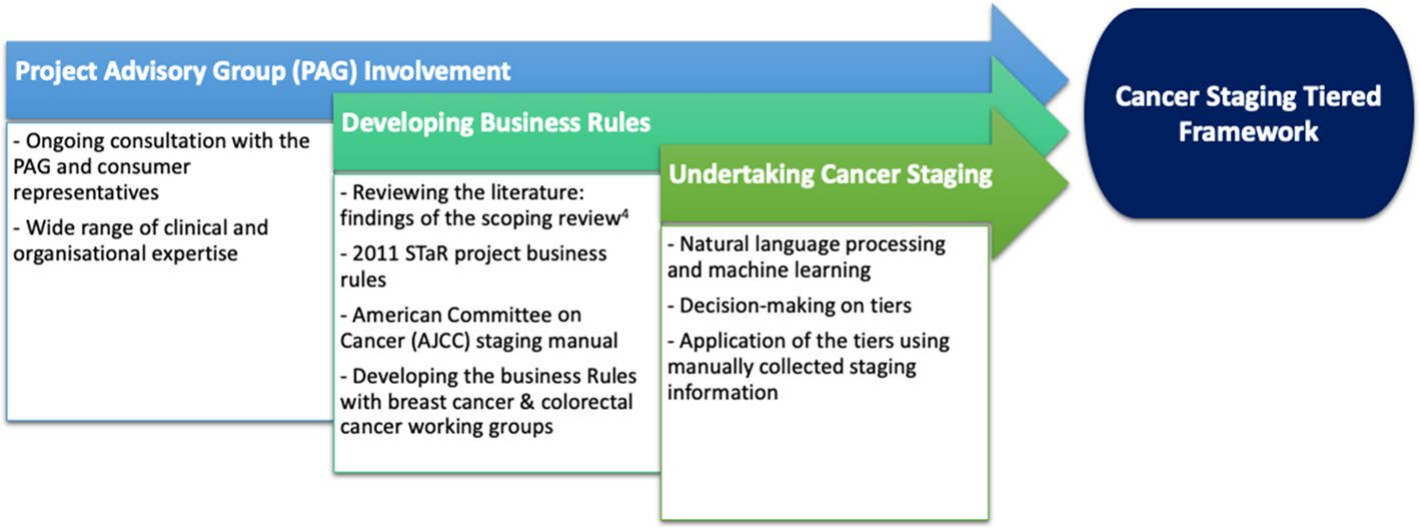
Cancer Staging Tiered Framework Development

The PAG collectively endorsed the AJCC TNM staging system as the most commonly used and established staging classification to be adopted by the WA Cancer Staging Project. Recognising the need for a tiered approach, the AJCC TNM system was seamlessly integrated into the framework. Additional insights into this developmental process can be found in our process evaluation [8]. Additionally, working groups, primarily consisting of clinical staff specialising in the specific cancer type for which cancer stage data were being collected, were actively involved in the development of the business rules at each tier (See Supplementary Material 1 for list of PAG and working group members). Figure 3 summarises this process.

Through discussions, both the PAG and working groups reached a consensus on the finalised cancer staging tiered framework’s criteria and business rules, ensuring they were comprehensive and applicable across different settings. The clinical working groups also focused on aligning the data with the AJCC TNM staging guidelines. This involved examining the codes from HMDC, ensuring that any assumptions made in the presence of specific HMDC codes remained in alignment with AJCC TNM rules. Decisions regarding assumptions and data alignment were made by consensus, further enhancing the framework’s robustness and applicability.

### Business rules at each tier

The following section details the business rules of the tiered framework as implemented in the WA Cancer Staging Project for collection of stage at diagnosis for the following tumour groups: breast cancer, colorectal cancer, and melanoma. The subsequent section will then discuss the quality implications and appropriate use of information at each tier, the process of transitioning between tiers, and future proofing the cancer staging tiered framework.

### Tier 1: Complete AJCC TNM

A Tier 1 classification is reached when complete AJCC TNM 8th edition can be collected, including all individual TNM values (category and subcategory), stage group, AJCC staging version, prefix classifications, tumour-specific fields (e.g., depth of invasion for colorectal cancer or hormone receptors status for breast cancer), and information related to the data source (e.g., MDT meeting notes) (see Fig. 2). Complete data is collected from trusted clinical sources, such as MDT software. In some cases, the complete TNM staging information may be available in the pathology report if the reporting pathologist has transferred across clinical staging information (such as the M category) from the EHR or MDM notes into the report. This is often reported using the “c” prefix to denote clinical staging information. See Table 1. This tier would also be suitable for including any prognostic staging scores. Additional information on the collection of prognostic staging is provided in the section titled ‘Future proofing the cancer staging tiered framework’. The recommended minimum dataset and data sources for each tier is available in Supplemental Material 2. Tier 1 data is suitable for clinical and epidemiological population-based analyses.

**Table 1:** Tier 1: Complete AJCC TNM (Colorectal Cancer)

Pathology Report with Clinical Staging Information			Stage Group
**Tumour (T)**	**Nodes (N)**	**Metastasis (M)**	
**p**T1	**p**N2	**c**M1a	Stage IVA

Based on the data available above, the final TNM derived is pT1N2 cM1a, Stage IVA. The bolded “p” and “c” represent “pathological” and “clinical” as the value’s data source

### Tier 2: registry-derived stage

The WACR derives RD-stage using available data sources where complete AJCC TNM cannot be collected. Within the cancer staging tiered framework, this is classified as Tier 2. Tier 2 builds and expands on Australian RD-stage methods (2011 STaR project), and the collection includes individual TNM values where possible, stage group, AJCC staging version, prefix classifications, tumour-specific fields, and the data source of TNM values (e.g., currently pathology reports or HMDC). In the WACR, Tier 2 leverages data supplementation from secondary data in the HMDC to make assumptions for nodal and distant metastases (see Table 2). HMDC is available from public and private facilities. To assign a stage group, assumptions are made that missing/not stated variables are considered absent (i.e., NX = N0 or MX = M0). For example, if a secondary metastatic disease code is present in HMDC (M = 1), stage group IV is assigned in colorectal cancer. In contrast, the absence of a secondary metastatic disease code (MX = M0) and a positive nodal involvement code (*N* = 1) would result in assigning stage group III. A limitation of Tier 2 collection is that the subcategory of nodal and distant metastases (i.e., N2a – four to six regional lymph nodes are positive in colorectal cancer) cannot be attained from ICD-10-AM coding in the HMDC. The ICD-10-AM coding only provides binary (yes/no) detail as to whether there are involved lymph nodes or secondary metastases present and does not provide the count of involved nodes or distant sites required for subcategory classification (see Table 3). This may potentially lead to under-staging; however, it still allows for the appropriate allocation of TNM within the main (umbrella) stage category. Since RD-stage is derived solely from this limited dataset and excludes additional factors like radiology reports and clinical correlation to assign stage, the data generated is recommended for primary use in population-based epidemiological studies.

**Table 2:** Tier 2: RD-Stage Only (Colorectal Cancer)

Pathology Report			Hospital Morbidity Data Collection		Stage Group
**Tumour (T)**	**Nodes (N)**	**Metastasis (M)**	**Nodes (N)**	**Metastasis (M)**	
pT1	pNX	pMX	N1	M0	RD-Stage III

Based on the data available above, the final TNM is T1N1M0, RD-Stage III, using the business rule assumptions that a positive lymph node ICD-code is equal to N1

**Table 3:** Tier 1 and 2 Comparison (Colorectal Cancer)

Pathology Report			Hospital Morbidity Data Collection		Tier 1 Stage Group	Tier 2 Stage Group
**Tumour (T)**	**Nodes (N)**	**Metastasis (M)**	**Nodes (N)**	**Metastasis (M)**		
pT1	**pN2a**	pMX	**N1**	M0	Stage IIIA	Stage III

Based on the data available above, the Tier 1 TNM derived is T1N2aM0, Stage IIIA. If pN2a was not available in the pathology report and HMDC is the only data source for nodal involvement, this will be categorised as Stage III (pT1 from pathology and N1M0 from HMDC) without the subcategory detail of Stage IIIA because information on the number of involved nodes is not available in HMDC. Nodal values are bolded for easy comparison

### Tier 3: pathology stage

Tier 3 (also “Pathology Stage”) collection is when Tiers 1 or 2 cannot be collected, and the WACR will collect the pathological stage described only in pathology reports. The collection includes individual pTNM scores where available, AJCC staging version, prefix classifications, and tumour-specific fields (see Table 4). In the WACR, not all patient events are recorded in HMDC, with the most common examples being patients who are treated privately or as an outpatient as they are not admitted to a public hospital. A limitation of relying solely on pathology stage is that the stage group may not accurately represent the complete extent of disease, potentially resulting in under-staging, especially when patients have clinically confirmed metastatic disease that is not reported in HMDC. Additionally, this approach is susceptible to bias since it predominantly includes patients undergoing surgical modalities. In instances where patients have received neoadjuvant therapy (NAT) before resection – an often preferred approach for certain cancer types – the pathology report may not explicitly indicate whether NAT was administered prior to the resection. This can occur due to the pathologist’s lack of awareness regarding the patient’s prior treatment or their failure to use the “yTNM” classification. The data generated through the Tier 3 method is recommended for epidemiological studies to offer a minimum level of insight into disease patterns and the population-level burden of the disease.

**Table 4:** Tier 3: Pathology Stage (Colorectal Cancer)

Pathology Report			Stage Group
**Tumour (T)**	**Nodes (N)**	**Metastasis (M)**	
**p**T1	**p**N2a	**p**MX	Stage IIIA

Based on the data available above, the final pathological stage is pT1N2aMX, Stage IIIA. This data does not consider admitted hospital data, clinical correlation with radiological imaging or MDT consultation. The bolded “p” represents “pathological” as the value’s data source

## Applying the cancer staging tiered framework: the WACR experience

Due to current data pipeline and infrastructure in the WACR, Tier 2 is typically achievable in most instances and is anticipated to remain the primary classification for most cases in the WACR well into the future. However, Tier 1 (Complete AJCC TNM) remains the gold standard for stage collection in the registry, should the data sources become available [8].

Table 5 illustrates the utilisation of the cancer staging tiered framework in the analysis of breast cancer, colorectal cancer, and melanoma cases collected in the WA Cancer Staging Project. The count of cases provided does not reflect the total population diagnosed during the specified years. Instead, it represents only a sample of cases extracted and manually collected for the development and validation of the NLP and ML staging algorithms. Within the melanoma cases sampled for 2019–2020 (*n* = 3049), the staging data exhibited varying levels of completeness across the defined tiers: Tier 1 was reached by only 1% of the cohort (*n* = 20), while Tier 2 displayed an expected 98% completion rate (*n* = 2981). Notably, Tier 3 showed no instances (0%, *n* = 4), and 1% of cases were categorised as unstageable due to the absence of available staging data (*n* = 44).

**Table 5:** Tiered staging framework application in melanoma, colorectal cancers, and breast cancers

**Tier**	**Melanoma** 2019–2020 (*n* = 3049) n (%)	**Colorectal Cancer** 2019 (*n* = 999) n (%)	**Breast Cancer** 2019 (*n* = 1712) n (%)
**Tier 1** *Complete AJCC TNM*	20 (1)	182 (18)	84 (5)
**Tier 2** *RD-Stage*	2981 (98)	598 (60)	1229 (72)
**Tier 3** *Pathology Stage*	4 (0)	21 (2)	18 (1)
**Unstageable**	44 (1)	198 (20)	381 (22)

In contrast, the staging data for colorectal cancer cases in 2019 (*n* = 999) showed more diverse results across tiers: Tier 1 was attained by 18% (*n* = 182), Tier 2 by 60% (*n* = 598), and Tier 3 by 2% (*n* = 21). Additionally, 20% of colorectal cases were classified as unstageable (*n* = 198). The staging data for breast cancer cases in the same year (*n* = 1712) most closely resembled the distribution observed in colorectal cancer cases. Specifically, Tier 1 was attained by 5% of cases (*n* = 84), Tier 2 by 72% (*n* = 1229), Tier 3 by 1% (*n* = 18). Notably, unstageable cases comprised 22% of the breast cancer cohort (*n* = 381).

The primary reason for tier distribution differences among the cancer types can be attributed to the distinct treatment approaches adopted for each group of cancers as recommended by the optimal care pathways [33]. As an example, stage data is frequently found in histopathology reports for cancer types that necessitate immediate resection post-diagnosis and have a higher incidence of early-stage cancer detection, like melanoma.

## Quality implications and appropriate use of staging data

The cancer staging tiered framework allows for the utilisation of staging data irrespective of its level of completeness, facilitating standardised reporting and comparability. This framework enables PBCRs to assess their cancer stage data in comparison to other PBCRs and exercise caution when interpreting data across various tiers. For instance, the WACR and VCR both collect staging data derived from multiple sources due to incomplete TNM staging information, aligning with Tier 2 – RD-Stage (Australasian Association of Cancer Registries: Australian PBCR Staging Assessment, unpublished). Implementation of the cancer staging tiered framework and reporting data alongside the tier may allow comparability of Tier 2 collected data while acknowledging the assumptions made to generate stage at diagnosis.

Data governance and quality control processes are essential for ensuring accurate, complete, and timely stage data collected by the WACR. Standardised protocols and best practices for data management play a crucial role in maintaining data integrity. The staging data, currently extracted from pathology reports and supplemented by HMDC where necessary, undergoes ad-hoc data quality validation. In this process, the WA Cancer Staging Project-collected data is compared with hospital clinical datasets containing staging data, particularly clinician-collected databases. As the WACR acquires additional existing stage datasets, further validation will take place. The staging data extracted by the NLP and ML models have also been validated against manually collected staging data, with conflicting cases undergoing further investigation by a cancer staging project officer for resolution. Additionally, oversight for HMDC is carried out by the Department of Health WA Data Quality Team, which executes formal validation processes on the dataset [34]. Protocols and procedures for the WA Cancer Staging Project were also developed and strictly followed, reinforcing the robustness of the data governance framework.

The depth and specificity of information available at each tier directly influence the accuracy and quality of cancer staging. When considering individual patients, the issue of data completeness arises because of variations in treatment pathways; not all patients undergo the same number of healthcare service interactions, resulting in differences in the availability of the detailed information required for stage calculation. Tier 1 achieves full data completeness by relying on comprehensive clinical data, offering the highest level of clinical accuracy in cancer staging as it directly draws from patient-specific clinical information, such as clinical and pathological correlation. In contrast, Tier 2, relies on assumptions about nodal and distant metastases based on secondary administrative data (hospital admitted patient data). While still providing reasonably accurate staging information, there may be some reduction in clinical accuracy. The accuracy of Tier 2 varies according to the cancer type; for example, lung cancer, with a higher incidence of metastatic disease at diagnosis [17], will likely yield more frequent metastatic disease codes in hospital admitted patient data compared to cancer types diagnosed at earlier stages. Lastly, Tier 3 exclusively utilises data from pathology, potentially resulting in an incomplete collection of the extent of disease. The clinical accuracy of this tier also varies according to the cancer type. For instance, earlier-stage cancers, such as melanoma [17], where surgical resection of the primary tumour is the initial treatment, are more likely to have pathological staging available. In contrast, cancers diagnosed at a later stage, where resection is not an option, may lack this information. The tiered approach balances clinical accuracy with data availability, ensuring that cancer staging remains relevant and informative across various data sources and contexts.

Despite potential concerns surrounding the conclusions drawn from the lower tier’s limited data, the insights it provides support a broader understanding of the population’s disease patterns, prevalence, and trends. It also aids in evaluating common risk factors and assessing overall disease burden at a population level, which can prove invaluable throughout public health planning, resource allocation, and policymaking decisions. Even at lower tiers, extracting staging data provides a valuable resource for epidemiological insights that would remain unknown if staging were completely unreported.

When analysing staging information across multiple tiers, the use of information should be targeted at the lower tier, as it offers a more conservative and standardised approach, minimising potential risks of misclassification associated with lower-tier data (for example, under-staging). For example, in a calendar year with staging data covering both Tier 1 and Tier 2 cases, the analyst should treat the entire cohort as Tier 2 and be used specifically for epidemiological analysis only, following the business rules for Tier 2. In certain cases, the staging data may be subject to separate analysis. For instance, if the public sector’s data contains only Tier 1 staging information due to the integration of an MDT meeting software in WA during a calendar year of data capture, this cohort could be analysed for clinical use, as well as for epidemiological use, while keeping the private sector data, which may not have adopted MDT meeting software, separated. The inclusion of the tier alongside staging details allows the analyst to interpret and utilise the information appropriately.

Future improvements in staging information and quality could involve integrating Tier 1 and 2 staging data with the routine collection of Patient Reported Outcomes Measures (PROMs) and Patient Reported Experience Measures (PREMs). PROMs capture patients' self-reported information on health-related aspects, such as symptoms and quality of life, while PREMs gather feedback on overall experiences with healthcare services, assessing satisfaction and perceptions of care [35]. Increasingly utilised in Australian registries, PROMs and PREMs have demonstrated benefits, including enhancing transparency of care, facilitating quality assessment, and enabling cost-effectiveness analysis [36]. These tools offer the potential for further comparisons with cancer treatments and cancer registries, informing healthcare delivery [36]. In the future, treatment variables could also be collected in PBCRs, enhancing the breadth and utility of the data. Integrating staging information with routine patient-reported data provides a more holistic understanding of both the clinical and patient-centred aspects of cancer care. Embedding this data in PBCRs not only supports continuous quality improvement by identifying areas for enhancement in clinical care and patient experiences but also creates opportunities for population research on the relationship between clinical outcomes and patient-reported data, contributing to evidence-based practices. The importance of collecting PROMs and PREMs as essential quality measures has been emphasised by the WA Cancer Staging Project’s PAG and is acknowledged in the literature, including the Australian Cancer Plan [37, 38].

## Transition between tiers

Lower tiers are only employed when capacity does not exist to collect the highest tier. A PBCR may collect staging information at all three tiers at any given time. As new data sources emerge, a PBCR might transition towards a higher tier for a greater proportion of cases. For instance, using the earlier example, if MDT meeting software is integrated across the public health sector in WA and linked into the WACR, this integration could facilitate a shift to Tier 1 collection for select cases. This shift is feasible due to the expectation that MDT meeting data will contain explicit clinical stage (cTNM). However, the private sector might lack this capacity, necessitating the continued use of Tier 2 or 3 collection.

## Future proofing the tiered staging framework

A tiered staging framework that standardises the collection of cancer stage in PBCRs not only enhances data consistency and comparability, but also ensures adaptability to improved access to more comprehensive data and updates in staging classifications. The framework presented in this paper captures anatomic stage information, offering insights into the extent and location of cancer within the body, as indicated by TNM. However, staging classifications continue to evolve in response to advancements in diagnostic and treatment technologies, alongside the discovery of clinically relevant tumour markers. There is a shift towards a more personalised approach that combines anatomic staging with biological and molecular markers [14]. This amalgamation aims to provide a more precise prognostic stage, with the ultimate goal of enhancing prognosis prediction and optimising treatment delivery, leading toward a more individualised approach to cancer management and better outcomes [14]. To incorporate prognostic staging into data collection, additional data variables are necessary. Typically, these data variables are available and summarised within data sources at a Tier 1 level, such as in clinical MDM notes. PBCRs in Australia have centred their efforts on the collection of anatomic stage data (Australasian Association of Cancer Registries: Australian PBCR Staging Assessment, unpublished). The data sources necessary for capturing these additional data variables for prognostic staging are either absent from their minimum datasets or have not yet been utilised to enhance their staging information. If a PBCR aims to integrate prognostic staging into its data collection, this expansion would logically align within a Tier 1, considering it has the technical capability to capture the necessary data variables within the existing structure. The tiered framework demonstrates a dynamic framework adaptable to changes in cancer staging classifications and data inputs.

Integration of high-quality data sources and improvement in data collection processes, as advances occur in their use and availability, is necessary for enhancing the collection of high tier cancer staging data. The inherent flexibility of the tiered framework, enabling registries with limited data (Tiers 2 or 3) to adapt their staging information collection according to available resources, provides a versatile approach to facilitate comparability between PBCRs with varied resourcing. This approach effectively mitigates the risk of data collection efforts being abandoned due to constraints related to data sources and infrastructure. The framework also motivates registries to continually refine their data collection procedures, particularly in recognising potential improvements, which may assist to futureproof the tiered staging framework. For instance, the four major pathology laboratories organisations in WA are actively improving the completeness of pathology data supplied to the WACR. This was an outcome of a pathology roadshow delivered by the WA Cancer Staging Project following on from a process evaluation recommendation [8]. Pathologists who are leading the way in the WA Cancer Staging Project’s pathology working group are currently assessing their compliance with structured reporting standards, resulting in the generation of more robust, complete, and comprehensive staging data. This enriched data will be funnelled into the WACR data pipeline for NLP and ML extraction, subsequently, channelling it into the tiered approach to result in a larger number of patients with complete data and minimising the number of unstageable cases.

The WACR is currently engaged in active discussions with the AACR, highlighting the WA experience (Australasian Association of Cancer Registries: Australian PBCR Staging Assessment, unpublished). As a result, the AACR is working towards establishing a comprehensive national tiered staging framework within Australia, taking into consideration a full assessment of the diverse data sources each state and territory PBCR has access to and their feasibility to collect each tier (Australasian Association of Cancer Registries: Australian PBCR Staging Assessment, unpublished). A national framework would enable all PBCRs to engage in the national collection of cancer stage data, thereby fostering national benchmarking. Our framework, as experienced in the WA setting, provides an estimation of the data sources required to achieve each tier and has assisted with establishing a national tiered staging framework. Assessing the feasibility of applying our business rules to other PBCRs in Australia and testing the adaptability and effectiveness of the framework, may prove valuable for advancing a national staging initiative.

The scalability and adaptability of the framework also make it valuable for international adoption. For instance, countries like Canada and the United Kingdom, which have provincial and regional cancer registries, respectively, could benefit from adopting the tiered staging framework [12, 39–41]. By learning from the WA experience, these countries could harmonise their data collection processes, allowing for more consistent and comparable data across provinces and regions. This adaptation would facilitate national benchmarking, improve the accuracy of cancer statistics, and foster international research collaborations with countries using similar frameworks.

The tiered staging framework’s structured methodology ensures adaptability to future updates in AJCC TNM staging classifications, advancements in templated and structured pathology reporting, and enhancements in data accessibility. This approach has the potential to harness all available data sources, address gaps in national cancer staging comparisons, and yield more accurate estimates of cancer stage at diagnosis, ultimately permitting assessment of patient outcomes and healthcare evaluation at the population level.

## Strengths and limitations of this study

A wide range of experts, including a PAG and expert clinical working groups, collaborated in developing the cancer staging tiered framework. To maintain continuous involvement and sustain interest among all stakeholders, the project team provided frequent updates through email and offered both in-person and online meetings. Despite the challenges posed by the COVID-19 pandemic and associated restrictions, regular online meetings were maintained. This inclusive approach ensured that both the PAG and expert clinical working groups had ample opportunities to contribute. Meeting minutes and various communication modes supported this effort, allowing us to leverage the substantial expertise of the project group and enrich collaboration.

The framework’s flexibility in accommodating diverse data collection approaches in recording stage at diagnosis recognises the need for tailored solutions. It allows long-term data integrity, interoperability, and standardisation for effective cancer-stage data management. Each tier within the framework incorporates adaptable business rules that can evolve alongside improved resources, data pipelines/sources, and technical capabilities. This is demonstrated in our paper through our current WACR data pipeline and infrastructure, where progression beyond Tier 2 or the expansion of stageable cases relies on improvements in data collection sources. Our ongoing efforts to address this challenge include initiatives such as the pathology roadshow aimed at enhancing report completeness and thereby mitigating data availability issues and technical constraints. However, data availability in WA remains a significant issue, with a lack of access to radiological imaging and MDM notes. These challenges are the main reason for adopting a tiered approach, which allows for flexibility and gradual improvement as more data sources become accessible.

The framework permits the utilisation of staging data regardless of its completeness, facilitates inter-PBCR data comparison, and distinguishes between clinical and epidemiological applications for better data interpretation. A limitation of the framework is that it requires additional resources and time for data collection and management, potentially posing logistical challenges for some PBCRs. For instance, implementing the framework means assigning tiers and storing related information within current databases. However, this challenge can be overcome by modifying existing infrastructure to include tier data variables in databases.

Regarding inter-PBCR data comparison, data following the AJCC TNM staging classification system can be reported as stage groups in each tier. While stage group can align with or be converted to other staging classification systems, there is a risk of misclassification and loss of granular stage information [4]. Despite these challenges, the data input into the tiered staging system still enables the formation of stage groups, facilitating inter-PBCR comparisons with PBCRs adopting different staging classifications.

A strength to highlight is the substantial funding and support received for the WA Cancer Staging Project, surpassing that of many other Australian PBCRs. The ongoing financial investment by the Cancer Network WA and expert involvement have positioned the project to address challenges effectively and achieve notable achievements in cancer staging data collection and management.

## Conclusion

The tiered cancer staging framework facilitates a consistent reporting format and promotes inclusive participation of all PBCRs, regardless of the extent of staging information they possess. It acknowledges data diversity for cancer stage collection among PBCRs, recognises that a one-size-fits-all approach is not suitable, and instead offers a pragmatic alternative to Tier 1 data collection – the gold-standard – where this is not attainable. Framework flexibility ensures both standardisation across PBCRs and practical utilisation of cancer stage data for optimising public health planning, including evaluating screening and early detection programs, monitoring treatment outcomes, guiding policy and funding decisions, and population health surveillance through epidemiological analyses. The cancer staging tiered framework, which was successfully implemented as part of the WA Cancer Staging Project now serves as a valuable resource for other PBCRs.

### Supplementary Information

Supplementary Material 1.

## Data Availability

The datasets generated and analysed during the current study are not publicly available as they are subject to strict data governance that restricts their use to authorised personnel for approved studies. Researchers wishing to access Western Australian Cancer Registry or other health data can apply via the WA Data Linkage System (https://www.datalinkageservices.health.wa.gov.au).

## Abbreviations

AACR: Australasian Association of Cancer Registries
AIHW: Australian Institute of Health and Welfare
AJCC: American Joint Committee on Cancer
CT: Computed Tomography
EHR: Electronic Health Record
HMDC: Hospital Morbidity Data Collection
ICD-10-AM: International Statistical Classification of Diseases and Related Health Problems, Tenth Revision, Australian Modification
MDM: Multidisciplinary Team Meeting
MDT: Multidisciplinary Team
ML: Machine Learning
MRI: Magnetic Resonance Imaging
NLP: Natural Language Processing
NAT: Neoadjuvant Therapy
PAG: Project Advisory Group
PBCR: Population-Based Cancer Registry
PET: Positron Emission Tomography
PREM: Patient Reported Experience Measure
PROM: Patient Reported Outcome Measure
REDCap: Research Electronic Data Capture
RD-Stage: Registry-Derived Stage
SEER: Surveillance, Epidemiology and End Results
STaR: Staging Treatment and Recurrence
TNM: Tumour-Node-Metastasis
WA: Western Australia
WACR: Western Australian Cancer Registry
