# Supplementary material for: Collecting routine and timely cancer stage at diagnosis by implementing a cancer staging tiered framework: the Western Australian Cancer Registry experience
Source: BMC Health Serv Res. 2024 Jun 28;24:770. doi: 10.1186/s12913-024-11224-4 (PMC11214229; doi:10.1186/s12913-024-11224-4)
Supplement: Supplementary file 1 — Supplementary Material 1. [file 12913_2024_11224_MOESM1_ESM.docx]

**Supplementary Material 1.**

Notes: WACR – Western Australian Cancer Registry; HIF – Health Information Fund; WA – Western Australia; DOH – Department of Health; WAKMAS – Western Australian Kirkbride Melanoma Advisory Service

***Figure 1 WA Cancer Staging Project: Project Advisory Group and Working Group members***

**Supplementary Material 2.**

Table 1. Cancer Staging Tiered Framework – Recommended Minimum Dataset

| **Data Element** | **Tier 1:**  ***Complete AJCC TNM*** | **Tier 2:**  ***Registry-Derived Stage*** | **Tier 3:**  ***Pathology Stage*** |
| --- | --- | --- | --- |
| **AJCC 8^th^ Edition Individual TNM Values** (Category/Subcategory) | Required | Not fully collected | Not fully collected |
| **Stage Group** | Required | Required | Required |
| **AJCC Staging Version** | Required | Required | Required |
| **Prefix Classifications** | Required | Collected where possible | Collected where possible |
| **Tumour-Specific Fields** (e.g., depth of invasion, tumour size, hormone receptor status, involved lymph nodes) | Collected where relevant | Collected where relevant | Collected where relevant |
| **Data Source**  (e.g., MDT Meeting Notes, Pathology Reports) | Complete clinical and pathology sources (MDT software, pathology reports, radiology) | Hospital admitted patient data, pathology reports | Pathology reports |
| **Prognostic Staging Scores**  (e.g., biological, and molecular markers) | Recommended | Not applicable | Not applicable |
| **Data Supplementation** | Not applicable | Hospital admitted patient data (assumptions made for nodal and distant metastases) | Not applicable |
| **Limitations** | None specified | Subcategory of nodal and distant metastases cannot be attained from hospital admitted patient data | Under-staging may occur, especially in cases of clinically confirmed metastatic disease not reported in hospital admitted patient data |
| **Primary Use** | Clinical and Epidemiological Population-Based Analyses | Primary use in Population-Based Epidemiological Studies | Primary use in Population-Based Epidemiological Studies |

Notes: MDT – Multidisciplinary Team.
